# Conflict of interest reporting in dentistry meta-analyses: A systematic review

**DOI:** 10.4317/jced.51225

**Published:** 2014-07-01

**Authors:** Mohammed M. Beyari, Dan Strain, Chuan S. Li, Hanadi A. Lamfon

**Affiliations:** 1 PhD, BDS, MSc, MFDRCSI. Umm Alqura University- Faculty of Dentistry; 2 BSc. Global Research Solutions Inc. Burlington, Ontario, Canada; 3 Umm Alqura University- Faculty of Dentistry

## Abstract

Objectives: The issue of reporting conflicts of interest (COI) in medical research has come under scrutiny over the past decade. Absolute transparency is important when dealing with conflicts of interest to provide readers with all essential information required to make an informative decision of the results. The key objective of this study was to examine the prevalence of reporting conflicts of interest in therapeutic dental meta-analyses of Randomized Control Trials (RCTs), and to investigate possible associations with other categorical variables. Study Design: We conducted an extensive literature search across multiple databases to search for relevant review articles for this study. We utilized pre-determined key words, and relied on three reviewers to test and review the use of a data extraction form that was used for the meta-analyses. Data regarding study characteristics, direction of results, and the significance of the results from each meta-analysis were extracted. Results: There were 129 meta-analyses used in this review, and the reporting on conflict of interest was low with only 50 (38.8%) of the articles possessing a conflict of interest statement (either confirming of denying COI). Of these 50 articles, there were only 4 (8%) studies that reported an actual conflict of interest. A statement of conflicts of interest was found in 29 (35.3%) of the papers that reported significant findings, whereas 35% of the papers that reported positive results reported on conflict of interest. Prior to 2009, only 17 (25%) papers reported conflicts of interest, but since 2009, 54.1% of papers collected had a conflict of interest statement. Conclusions: Meta-analyses published in the field of dentistry do not routinely report author conflicts of interest. Although few conflicts appear to exist, the field of dentistry should continue to ensure that best evidence reports provide clear and transparent reporting of potential conflicts of interest in academic journals.

** Key words:**Dentistry, dentition, meta-analysis, quantitative review.

## Introduction

In recent years, there has been a great deal of scrutiny directed at scientific research, and medical journals regarding biased scientific findings, and conflicts of interest (1). The role of industry in medical research has grown tremendously, blurring the lines between industry and medical research, leaving the topic highly debated (2). In 2009, The World Association of Medical Editors [WAME], one of the more active members of this debate, stressed the importance of transparency in regards to personal interests (2). While not pushing for a universal standard, WAME did provide editors with an expanded version of what it means to have personal interests, and what they think should be disclosed while performing medical trials (2).

To perform dental research and multi-clinical medical trials, it is absolutely essential that there is a certain amount of cooperation between physicians, dentists, and industry (3). Industry has the ability to allocate funds within the research sector, thus creating potential conflicts of interest. A conflict of interest is a set of conditions in which professional judgment about a primary interest [such as a patient’s welfare or the validity of research] is unduly influenced by a secondary interest [such as financial gain] (1). Companies that produce drugs or medical devices are often susceptible to conflicts of interest, as their funding can impact reporting of the results, and general design and outcomes of medical research trials (3). This is illustrated by the finding that industry-sponsored studies more frequently express positive results towards that industry or product, than do studies that have alternative sources of funding (4,5). For these reasons, transparency in reporting conflict of interest in medical journals has been a very important topic of discussion over the past few years. With the increased availability of journals to the public, and the plethora of media attention that comes with a delayed admittance of conflict of interest in a high-impact study, there has never been more pressure on authors to reveal any personal bias. The field of dentistry does not currently have an organized ethics committee to aid in addressing issues including conflict of interest, and a recent survey of dentists reported that half of respondents were not aware of any guidelines regarding conflict of interest in dentistry (6). This can lead to severe conflicts of interest not being reported, and ultimately, can challenge the public’s confidence in regards to scientific findings (1).

The primary objective of this systematic review is to assess the prevalence of conflicts of interest and conflict of interest statements in therapeutic meta-analyses of randomized controlled trials [RCTs] on dental-related topics. Secondary objectives seek to examine how COI can affect the significance, and the direction of the results, and how trends of conflicts of interest reporting have changed over the past decade.

## Material and Methods 

- Study Eligibility Criteria

All systematic reviews with a meta-analysis component, containing at least one RCT, and published between January of 2000 and June of 2012 were included for review. Literature reviews and systematic reviews published within the Cochrane Database of Systematic Reviews [the gold standard of meta-analyses] were excluded.

- Information Sources

We conducted a comprehensive literature search to identify relevant studies to be included. The search was applied electronically through the following bibliographic databases via OVID at the University of Toronto: Medline [2000-June 2012: In Process and Other Non-Indexed Citations], EMBASE [2000 to June 2012], and PsychINFO [2000 to June 2012]. The same search was applied to the CENTRAL database [via Cochrane]. No limits were applied for language, and all non-English articles were translated. The last search was run on June 3, 2012.

- Search

**Figure 1:**
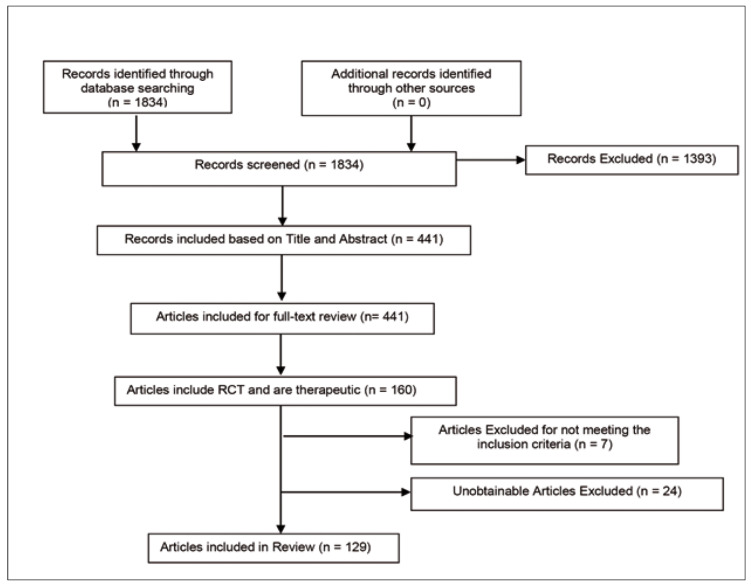
Study Flow Diagram.

With the collaboration of a librarian at the University of Toronto, a comprehensive search strategy was developed for each of the aforementioned electronic databases. The following search terms were used to search all databases and trial registers: dentistry, dentition, meta-analysis, quantitative review. Refer to figure 1 for a sample search strategy.

- Study Selection

Our study selection was conducted independently by one reviewer [MER] and revised by another reviewer [CSL]. In the first stage the full yield of the search was reviewed and potentially eligible articles were identified based on either their title or abstract. Following the initial screening step, results from both reviewers were pooled. Following the deletion of duplicates, full-text reviews of these results were, again, conducted by one author [MER] and reviewed by another [CSL]. Agreement between the two reviewers following this stage was calculated and presented as a kappa value. Disagreements between the two reviewers were resolved by consensus and discussion with the senior authors [HL and MB].

- Data Extraction

A data extraction form was designed and pilot-tested on ten randomly selected studies by three reviewers [MB, CSL, DS] in order to ensure the standardization of the data collection process. The three reviewers independently and in duplicate extracted data regarding the study characteristics, the significance and direction of the results, and the potential conflicts of interest. All disagreements were resolved by the consensus of the reviewers, in correspondence with the senior authors [HL and MB], following which the level of agreement was calculated and presented as a kappa value. All collected data were stored in a Microsoft Excel™ file.

- Data Items

Information was extracted from the included studies on:

1. Study characteristics, including the year of publication, the journal in which the study was published, the

country of origin, the dental area of study, and the number of included studies.

2. The presence of potential conflicts of interest.

3. The significance and direction of the results

- Data Analysis

Data were analyzed using Microsoft Excel 2010 [Microsoft Corp., Redmond, WA]. Descriptive statistics [percentages, means] were calculated to describe the demographic characteristics of the studies and COI reporting trends. Chi-square tests were performed to assess for significant associations at a significance level of *p*<0.05. A linear regression was also used to inspect possible associations between the number of primary studies used and the year in which the paper was published.

## Results

There were a total of 129 meta-analyses collected for the purpose of this study, dating back to 2000 ( Table 1). From the studies collected, the two major contributing continents were Europe and North America, which contributed 70% [90] of the total papers. Of those 129 collected, 38.8% or 50 studies referenced the presence or absence of a conflict of interest. As a result, 61.2% of the meta-analyses that were collected for this paper did not have adequate disclosure and did not report a conflict of interest statement. Moreover, it was reported that only 19 meta-analyses cited RCTs within them that confirmed to have a COI.

**Table 1:**
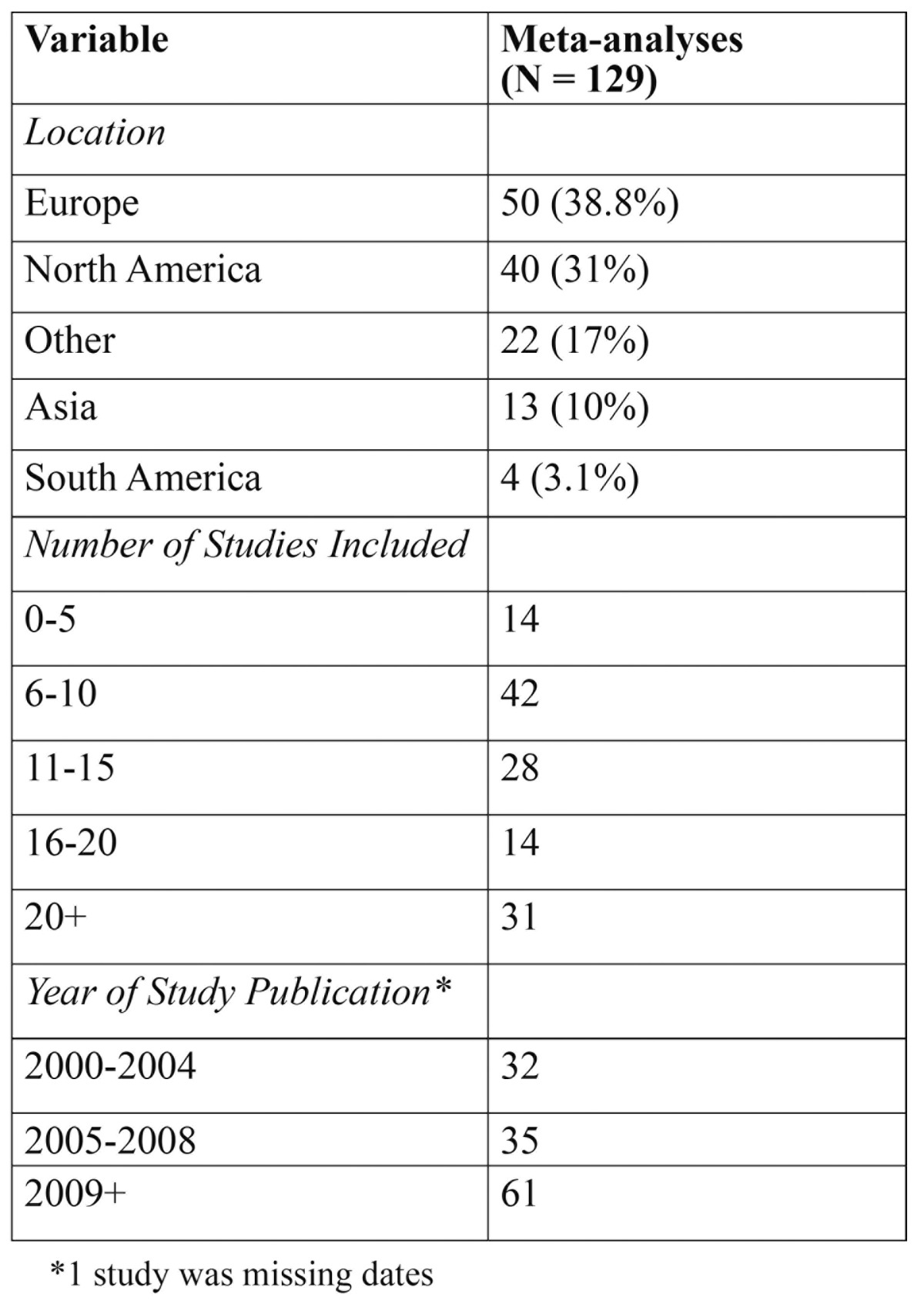
Characteristics of Included Studies.

Of the 50 cases that had a conflict of interest statement, only 4 [8%] had absolute conflicts of interest present.

When examining reported conflicts of interest statements, we explored its possible associations with significance, and direction of results that were found. Significant findings were reported in 82 [63.6%] of the meta-analyses. Similarly, of the 50 papers that had conflict of interest statements, 29 [58%] were of significance. Consequently, only approximately 35% of papers with significant results mentioned conflict of interest, and as a result, we found no association between the reporting of conflict of interest and the significance of the findings [*p*=0.29]. Examining the four cases of actual conflict of interest, 75%, or, 3 out of the 4 cases, reported significant findings, however, further research is needed as the low number of events does not allow for firm conclusions.

**Figure 2:**
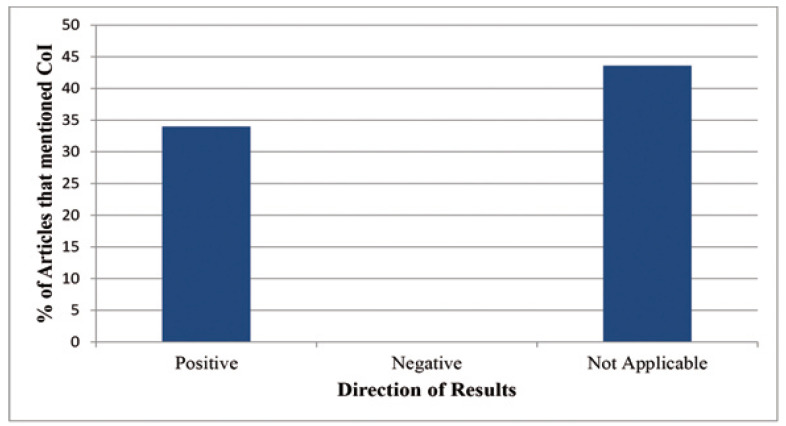
Percentage of articles mentioning COI vs. Direction of Results: The percentage of articles that reported on COI based on the direction of results.

The direction of the results is also vital to the analysis when looking for trends in conflict of interest. A total of 55 [42.6%] cases reported positive results, however, only 1 of the 129 meta-analyses collected reported an overall negative result. A large number of the papers [56.6%] did not present findings with a clear direction. Of those studies that reported positive results, only 35% of them had a statement disclosing potential conflicts of interest, resulting in no noteworthy association between COI statement reporting, and the direction of the results [*p*=0.47] (Fig. 2). The 4 papers that were determined to have absolute conflicts of interest included 2 reporting positive results, and 2 in which findings were not applicable.

**Figure 3:**
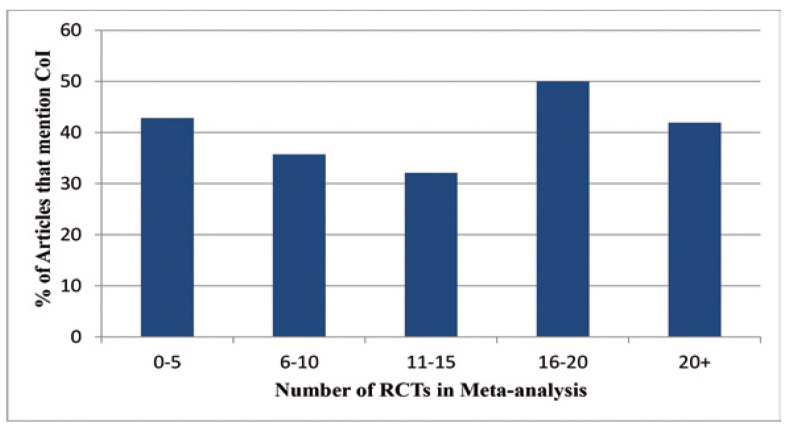
Percentage of articles that report on COI vs. Number of RCTs in each Meta-analysis: The percentage of articles that mentioned COI based on the number of RCTs that were used in the meta-analysis.

**Figure 4:**
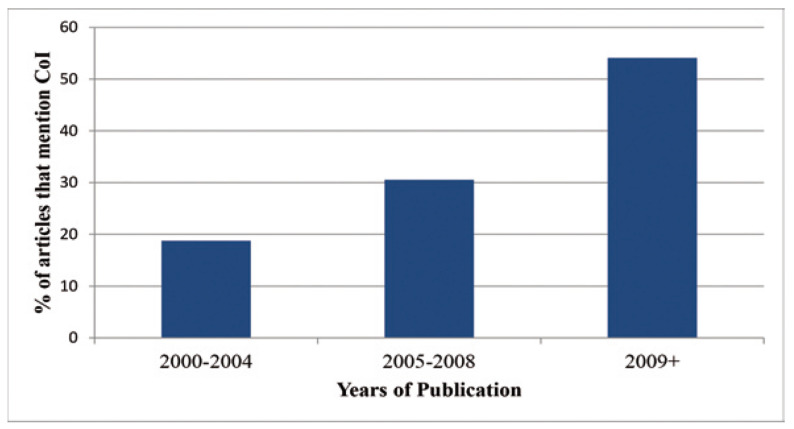
Percentage of articles that mentioned COI: This figure shows the percentage of articles that report on COI based on each group of years.

Figure 3 shows the prevalence of reporting on conflicts of interest and number of primary articles used in each meta-analysis. A relatively even distribution is seen, and there is no significant association of reporting COI statements and the number of primary articles used. The possibility of an association between papers reporting on conflict of interest and the year in which they were published was also explored. The years in question were separated into groups of 4-5 years, and were measured as a part of these groups. Keeping in mind that one study was missing dates, of the 129 total studies, 32 were published between 2000 and 2004, 35 between 2005 and 2008, and 61 of the papers were published after 2009. The papers published prior to 2009 had comparable results, with 18.8% of papers reporting conflict of interest statements prior to 2005, and 30.1% reporting them between 2005 and 2008. However, when studies conducted after 2009 were examined, it was found that more than half [54.1%] of papers reported on issues of conflict of interest, a significant increase [*p*=0.002] (Fig. 4).

## Discussion

While previous studies have shown that the presence of conflict of interest tends to affect the significance or direction of results reported, in our study, no such associations were found (4). The primary reason that we were unable to report on this is due to the small number of papers that were determined to have a conflict of interest. This is a positive result for the field of dentistry, as it demonstrated that COI is not a major predictor of study outcome in this field. Due to the small number of papers with an actual conflict of interest, the 50 papers that reported conflict of interest statements became the primary focus of this study. In regards to significance of results, the papers that reported significant findings only accounted for approximately 35% of the meta-analyses that had conflict of interest statements. Looking at the number of cases that did not report on whether or not there was a potential conflict of interest, we find that two thirds of the studies [66.25%] reported statistically significant findings. Many of these papers may have had no conflict of interest at all, which may be why there is no COI statement, but in the interest of transparency of personal interests, it would be ideal for articles to report on conflict of interest whether there is one or not. In doing so, they would help readers to avoid speculating when nothing is reported on conflict on interest in either direction. When looking at the association between direction of results and prevalence of conflict of interest statements, we also found no significant correlation between these two variables. This, in large part, can be attributed to the lack of studies that were extracted with results applicable to direction. This large number of non-directional studies can be credited to the design of the studies that were sampled, but one could also argue the possibility that authors may potentially report their results as not applicable to avoid reporting negative results. In this situation, declaration of conflict of interest statements, as well as funding sources would aid readers in making a judgement for themselves regarding bias of the results.

We also explored the possibility of associations between conflict of interest statements and number of primary studies used in the meta-analysis, as well as between number of primary studies and the year in which the paper was published. In both circumstances, we found there to be no significant correlation between the two variables, indicating that the number of primary studies might not affect the authors’ tendency to report on conflict of interest.

A significant association was noted for the years in which the paper had been published compared to whether or not a conflict of interest statement was utilized. We found that there was a significant positive correlation between publication date and mentioning conflict of interest; papers published more recently were more likely to report on the presence or absence of a conflict of interest. This is an important finding, as it helps to demonstrate that in recent years, authors are feeling increased pressure regarding mentioning potential conflicts of interest or there is more conflict of interest to report. This increase could also be a result of dental journals becoming stricter on issues of disclosure, and enforcing policies on reporting more diligently. Regardless, having an increase of authors reporting conflict of interest statements is imperative moving forward (7). It has been suggested by Faggion (7) that dental journals adopt the format introduced by the International Committee of Medical Journal Editors [ICMJE] regarding the disclosure of personal interests. Utilizing the form issued by the ICMJE would allow for a standardized form of reporting conflicting interests that could be compared across modalities, and more importantly, push for complete transparency when it comes to conflicts of interest (7).

A noted strength of the current study is the number of articles that were included. The inclusion of 129 meta-analyses specifically in the field of dental research helps to demonstrate the extent of the concerns regarding reporting conflict of interest statements. Furthermore, it allowed for data collection across numerous dental modalities, thus preventing the possibility of the results being skewed by certain practices or journals. Another strength of this paper is that all articles that were used in this review have been published since 2000, allowing for a more recent review of trends of conflicts of interest in dental journals.

An apparent limitation of this review is that funding sources were not examined and extracted during the collection of data. By examining the sources for which studies are receiving their funding, we could identify which studies have conflicts of interest more efficiently. Furthermore, by identifying studies that are industry sponsored, we could increase our understanding of the prevalence of conflict of interest statements by those who clearly have personal interests involved.

 A study by Roseman and colleagues (8) investigated the prevalence in which meta-analyses published in high-impact medical journals report on funding sources and conflicts of interest of RCTs that they include. They report that in general, meta-analyses rarely report on COI and funding sources, citing that meta-analyses have no obligation in many high-profile journals to report on conflicts of interest of their included studies (8). These findings are similar to those of the present study, and indicate that the concern of lack of conflict of interest reporting is not exclusive to the field of dentistry. The field of orthopaedics, for example, has a huge prevalence of COI, with some studies calling for a change in relationship with industry, as they are becoming too powerful within orthopaedic trials (9,10).

While issues of conflict of interest have been going on for many years, only recently has the definition been catching up to the ever evolving meaning of this maxim (1). According to WAME, there are 5 ways in which someone can have conflicting interests in a research study including, financial ties, academic commitments, personal relationships, political or religious beliefs, and lastly, institution affiliations (2). The results of this study illustrate quite clearly that there is still substantial variability when it comes to the reporting of conflict of interest statements in dentistry journals. Although meta-analyses are not clinical trials in themselves, they can have a significant impact on the decisions medical professionals and patients make regarding treatments, procedures and new technologies. It is vital for dentistry journals to standardize reporting of conflicts of interest to maximize the transparency and integrity of the research.
